# Online Health Information Wants of Caregivers for Persons With Dementia in Social Media

**DOI:** 10.1093/geroni/igab046.2431

**Published:** 2021-12-17

**Authors:** Alison Tang, Jung Kwak, Bo Xie, Lu Xiao, Sucheta Lahiri, Olivia Flynn, Avinav Murugadass

**Affiliations:** 1 The University of Texas at Austin, Austin, Texas, United States; 2 Syracuse University, Syracuse University, New York, United States; 3 Syracuse University, Syracuse, New York, United States; 4 Syracuse University, Syrcause, New York, United States

## Abstract

Family caregivers of persons with dementia (PWDs) use social media to obtain and share health information. Yet relatively little is known about the types of information that these caregivers share. We address this gap by analyzing caregivers’ posts (N = 401) in two subreddits, r/Alzheimers and r/dementia, which represent two subgroups on the social media platform Reddit. Our research questions were as follows: (1) What are these caregivers’ main purposes in posting? (2) What types of health information do the caregivers exchange online? (3) What are the characteristics of online posts that receive the caregivers’ greatest attention? Content and correlational analyses revealed that over half of users (57%) posted regarding specific types of health information that they desired to seek and share, whereas the remaining users posted information for sharing only. The health information most commonly posted fell into the following three categories: psychosocial health information, information about patients’ daily care, and characteristics of health conditions. The more health information types that a post contained, the greater the number of people who participated in the subreddit discussions. Further research should examine how social media meet PWD caregivers’ needs for information support.

